# Worm control practice against gastro-intestinal parasites in Norwegian sheep and goat flocks

**DOI:** 10.1186/1751-0147-53-29

**Published:** 2011-05-13

**Authors:** Atle VM Domke, Christophe Chartier, Bjørn Gjerde, Nils Leine, Synnøve Vatn, Olav Østerås, Snorre Stuen

**Affiliations:** 1 Norwegian School of Veterinary Science, Sandnes, Norway; 2 National College of Veterinary Medicine, Food Science and Engineering-ONIRIS, Nantes, France; 3 Norwegian School of Veterinary Science, Oslo, Norway; 4 Norwegian Goat Health Service, Oslo, Norway; 5 Norwegian Sheep Health Service, Animalia, Oslo, Norway

## Abstract

**Background:**

Anthelmintic treatment is the most common way of controlling nematode infections in ruminants. However, several countries have reported anthelmintic resistance (AR), representing a limitation for sustainable small ruminant production. The knowledge regarding worm control management represents a baseline to develop a guideline for preventing AR. The aim of the present study was therefore to improve our knowledge about the worm control practices in small ruminant flocks in Norway.

**Methods:**

A questionnaire survey regarding worm control practices was performed in small ruminant flocks in Norway. Flocks were selected from the three main areas of small ruminant farming, i.e. the coastal, inland and northern areas. A total of 825 questionnaires, comprising 587 sheep flocks (return rate of 51.3%) and 238 goat flocks (52.6%) were included.

**Results:**

The results indicated that visual appraisal of individual weight was the most common means of estimating the anthelmintic dose used in sheep (78.6%) and goat (85.1%) flocks. The mean yearly drenching rate in lambs and ewes were 2.5 ± 1.7 and 1.9 ± 1.1, respectively, whereas it was 1.0 (once a year) in goats. However, these figures were higher in sheep in the coastal area with a rate of 3.4 and 2.2 in lambs and ewes, respectively. Benzimidazoles were the predominant anthelmintic class used in sheep flocks (64.9% in 2007), whereas benzimidazoles and macrocyclic lactones were both equally used in dairy goat flocks. In the period of 2005-2007, 46.3% of the sheep flocks never changed the anthelmintic class. The dose and move strategy was practiced in 33.2% of the sheep flocks.

**Conclusions:**

The present study showed that inaccurate weight calculation gives a risk of under-dosing in over 90% of the sheep and goat flocks in Norway. Taken together with a high treatment frequency in lambs, a lack of anthelmintic class rotation and the common use of a dose-and-move strategy, a real danger for development of anthelmintic resistance (AR) seems to exist in Norwegian sheep and goat flocks. This risk seems particularly high in coastal areas where high treatment frequencies in lambs were recorded.

## Background

Nematode parasitic disease remains one of the greatest limiting factors in successful, sustainable ruminant livestock production worldwide. Currently, the control of nematode infections still relies mainly on the use of effective anthelmintics, which often represent the simpler, safer and cheaper option [1]. However, anthelmintic-resistant (AR) nematodes are now recognised as an important threat to the productivity and welfare of sheep in many parts of the world, including Europe [2]. The profitability and sustainability of the goat industry are also seriously threatened by rapid development of AR [3].

Twenty years ago, AR was a devastating problem only of the Southern Hemisphere, but now it has been recognized as a global concern. In some countries of Northern Europe, resistance to benzimidazoles has been found in up to 80% of the goat flocks, even in a context of a rather limited drenching frequency [4], and resistance to two, or even all three, major classes of anthelmintics has been recorded for goats in France and for sheep in Scotland [5-7]. However, the situation may be different in some other European countries such as Sweden, Germany and Slovakia, where AR have been detected years ago at a rather low frequency [8-10]. Different situations may also be encountered regarding the nematode species involved and the intensity of infection. Severe clinical reports of AR in small ruminants with very high worm burdens have been made with *Haemonchus contortus, *especially in tropical areas, whereas subclinical cases were more frequently described with the common abomasal (*Teladorsagia circumcincta*) and intestinal (*Trichostrongylus colubriformis*) worms under temperate areas. However, although less severe than under the tropics, drug resistance issue is in the UK considered a threat to the economical sustainability of sheep production [11].

In Norway, only benzimidazoles and macrocyclic lactones are licenced for use in sheep and goats. The release of the amino-acetonitrile derivative (AAD) drug, monepantel, in 2010 represents the first new anthelmintic class since the launch of ivermectin in the early 1980s for use in sheep, but this drug is not yet licenced in Norway.

The AR situation in Norway is, however, not well documented. Only sporadic cases of AR in small ruminant flocks have so far been reported in Norway [12].

The main factors for selection for anthelmintic resistance are: i) the application of anthelmintics in a situation where parasites have a small refugia [13,14], ii) a high treatment frequency [15], iii) under-dosing [16,17], and iv) the use of the same anthelmintic class over several years [17]. These factors, alone or in combination, together with certain types of farm management can accelerate the development of AR [1,18,19].

Norway has a great variability in climatic and geographical conditions, which influences the farm management. The inland and northern areas have an alpine climate in contrast to the subalpine climate in the coastal lowland area [20]. There is approximately one million winter fed sheep in Norway, with an average flock size of 66 animals [21]. Ewes and lambs are normally put on fenced spring pastures one or more weeks after lambing. After a few weeks, the ewes and lambs are moved to common rangeland pastures in the mountains or forests. The densities of sheep on mountain pastures in Norway vary between 10 and 80 animals per km^2 ^[22]. It is estimated that more than 2/3 of all sheep are moved to grazing on mountain or forest pastures from June-July until August-September [23]. In contrast, dairy goats, which in Norway represent a total number of approximately 40 000 animals older than one year, with an average flock size of 83 animals, are normally on pastures close to the farm during the whole grazing period [21]. However, in some areas of Norway the use of mountain dairy farming during summer is still practiced [24]. In addition, there are approximately 4000 fiber (non-dairy) goats in Norway with an average flock size of 6.5 animals.

Surveys based on questionnaires regarding worm control practices in small ruminants in Europe have previously been done in Denmark [25,26], the United Kingdom [27-30], France [31] and the Slovak Republic[32]. In Norway, a similar survey has not been performed. The aim of the present study was therefore to improve our knowledge about the worm control practices in small ruminant flocks in Norway. The objective was to focus more specifically on the anthelmintic usage and to examine to what extent the risk practices for developing AR were encountered in these flocks.

## Methods

### Questionnaire

In the autumn of 2007, a questionnaire was sent to Norwegian sheep and goat farmers in order to collect information concerning which gastrointestinal parasite control measures had been used during 2005-2007, such as pastures management, deworming practises and the use of anthelmintics. The flocks were selected from the Norwegian governmental list of farms that apply for production subsidies [21]. Before this, two small pilot studies with a limited number of farmers involved were performed in order to avoid misunderstandings and misinterpretations of the final questionnaire. The questionnaire was comprised in two sections. The first section was related to main characteristics of the farm management such as flock size, breed, grazing conditions and housing time. The second section was dedicated to the use of pastures and the anthelmintic parasite control practice. Questions regarding clinical signs linked to parasitism, time and reason for anthelmintic treatment, anthelmintic products used in the years 2005 to 2007, dose determination, mode of application and the source of information regarding worm control, were included.

### Selection procedures

**Figure 1:**
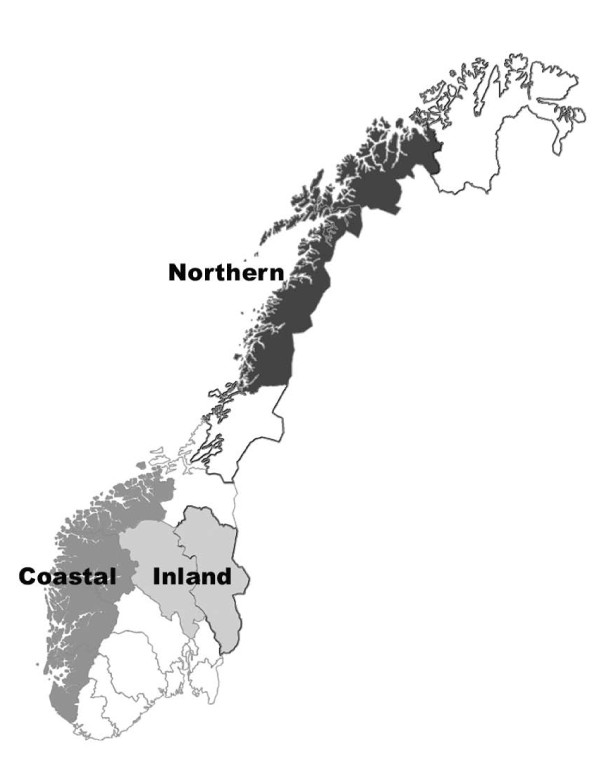
**Map of Norway showing the three geographical areas represented in the survey**.

Flocks were selected from the three main areas of small ruminant farming; i.e., Rogaland, Hordaland, Sogn og Fjordane and Møre og Romsdal counties in western Norway (the coastal area), Hedmark, Oppland and Telemark counties in south-eastern Norway (the inland area), and Nordland and Troms counties in northern Norway (the northern area) (Figure 1). These three areas also represent the main types of grazing and farm management in Norway. In each area, flocks from a minimum of three municipalities were selected.

Only sheep farms with a flock size larger than 20 winterfed ewes were included, i.e., 9.8% of the total number of flocks with more than 20 ewes in Norway [21]. A total of 1145 sheep flocks were randomly selected, including 522 flocks with a flock size of 20-100 ewes (selection rate of 5%) and 623 flocks with more than 100 ewes (selection rate of 50%). In addition, dairy goat farms with more than 24 goats and fiber goat (non-dairy) flocks with more than 6 goats were included. In total, 379 farms with dairy goats and 73 farms with fiber goats were selected, representing 100% of all goat farms fulfilling the inclusion criteria. The questionnaire was not anonymous. Farmers that did not respond to the questionnaire within one month, were reminded once.

### Statistical analysis

SPSS (SPSS Inc. Chicago, Version 16.0) and Excel 2003 (Microsoft Inc.) were used for statistical calculations. The Kruskal-Wallis ANOVA was used for calculating the significant differences regarding number of anthelmintic treatments in different areas of Norway. Chi-square analyses were used for group differences using SAS (SAS Institute Inc., Cary, NC).

## Results

A total of 825 questionnaires (51.6%) were returned, representing 587 sheep flocks (51.3% of those selected) and 238 goat flocks (52.6%). These flocks represented 74472 winter fed sheep, 20466 dairy goats and 262 fiber goats. All sheep flocks were considered together, independent of flock size. Fiber goats were represented in 14 (5.9%) of the goat flocks and were grouped together with the dairy goats in the calculations. Seventy (8.5%) of the flocks had both sheep and goats, but each of these was treated as either a sheep or a goat flock in the survey.

### 1. Sheep flocks

#### 1.1 General information

The three main sheep breeds represented in the sampled farms were: the Norwegian White Sheep ("Norsk Kvit Sau") with 76.2% of all sheep flocks, the Old Norwegian Short Tail ("Spelsau") with 11.4% of the flocks, and the Norwegian Feral Sheep ("Villsau") with 4.2% of the flocks (data not shown). This distribution reflects the Norwegian sheep population well [21]. Lambs together with their ewes were turned out on pasture in April/May in the coastal area and in May/June in the northern and inland area. Average lamb age at turnout was 2-3 weeks (Table 1). Lambs had a grazing period lasting 6-8 months in the coastal area and 6-7 months in the inland and northern areas. During the summer period, 68.5% of the sheep flocks used a mountain or forest pasture. In 68.6% of the sheep flocks, grazing together with cattle both at home pasture and on the mountain/forest pasture were practiced, while less than 4% of the sheep flocks co-grazed with goats only. The local veterinarian (56.0%), agriculture magazines (22.2%) and other farmers (18.9%) were the three most important sources of information regarding worm control practice (data not shown).

**Table 1:** General management characteristics of Norwegian sheep and goat flocks in present survey.

	Sheep	Goats
Number of flocks (%)	587 (100)	238 (100)
Mean flock size ( ± SD) (range)	126.3 ± 74.4 (20-500)	95.0 ± 45.1 (12-300)
Number of farms with organic farming (%)	29 (5.2%)	18 (7.6%)
Mean turn out time on pasture	Early May	Mid-May
Mean range of age in lambs/kids at turn out (weeks)	2 - 3	>12
Mean housing time	Late September	Mid-September
Practicing dose-and-move (percentage)	212 (35.8)	5 (2.1)

#### 1.2 Drenching time

In 57.4% of the flocks the timing and frequency of the anthelmintic treatments against gastro-intestinal parasites were based only on previously established routines and experience, together with regularity and when housing the animals (Table 2). The dose and move strategy was practiced in 33.2% of the sheep flocks.

**Table 2:** Main determination of time and routines for anthelmintics drenching in Norwegian sheep and goat flocks (frequency and percentage).

			Sheep		Goats	
			
			n	%	n	%
Determination of treatment time	Sheep flocks (*n *= 587)	Pasture rotation	195	33.2		
		Only at housing	51	8.7		
		Faecal egg count	0	0.0		
		Clinical signs/scouring	4	0.7		
		Combination^a^	337	57.4		
	Goat flocks (*n *= 235)	Non lactating period			199	84.7
		Pasturing			21	8.9
		Faecal egg count			1	0.4
		No treatment			14	6.0
Calculation or estimation of weight		Individual weight	12	2.1	1	0.8
		Weighing (heaviest animal)	35	6.2	2	1.7
		Weighting (mean sized animal)	69	12.2	5	4.1
		Visual appraisal	444	78.6	103	85.1
		No estimation^b^	5	0.9	10	8.3
		Total	565		121	
Control of drenching gun		1 per flock treatment	225	41.4	94	51.9
		2 per flock treatment	90	16.6	11	6.1
		>2 per flock treatment	81	14.9	7	3.9
		Never	147	27.1	69	38.1
		Total	543		181	

^a ^Combination: Treatment time based on previously established routines and experience, together with regularity and housing.

^b^done by veterinarian.

#### 1.3 Dose-estimation

In 78.6% of the sheep flocks the anthelmintic dose was determined by visual appraisal of the weight of the heaviest lamb or ewe before the start of the drenching operation. In 27.1% of the sheep flocks, the farmers never checked the accuracy of their drench guns when giving their animals an anthelmintic treatment.

#### 1.4 Drenching frequency

The mean yearly drenching rate in lambs and ewes were 2.5 ± 1.7 and 1.9 ± 1.1, respectively (Table 3). The mean drenching rate of lambs in the coastal, inland and northern area was 3.4, 2.0 and 1.3 times per year, respectively. For ewes the drenching rates in the same areas were 2.2, 1.6 and 1.5 times per year, respectively. In 8.7% of the sheep flocks, the treatment against gastro-intestinal parasites was given only at housing in the autumn (Table 2). In contrast, in ten flocks (1.7%) the number of annual treatments for lambs was ≥7. Nine of these ten flocks were located in Rogaland County. In the coastal area, lambs were treated more than three times per year in 45.9% of the flocks (Table 3). This was a significantly higher number of treatments compared to the two other areas (p < 0.001).

**Table 3:** Distribution of flocks according to the annual number of treatments against gastro-intestinal parasites in lambs and ewes in the different areas (percentage) of Norway.

Number of treatments		n	Coastal	Inland	Northern	Total
Lambs	1	144	13.9^a^	33.0	47.6	26.5
	2	174	19.5^a^	44.3	47.6	32.0
	3	87	20.7^a^	14.7	3.6	16.0
	>3	139	45.9^a^	8.0	1.2	25.5
	Mean^b^		3.4	2.0	1.3	2.5
Ewes	1	202	24.3	38.9	51.0	36.4
	2	246	44.7	52.2	43.8	44.3
	3	72	20.4^a^	6.7	5.2	13.0
	>3	35	10.6^a^	2.2	0.0	6.3
	Mean		2.2^a^	1.6	1.5	1.9

The mean number is also calculated for each area.

^a ^superscripts indicate p < 0.001 (Kruskal-Wallis) between this area compared with the other areas in the same line.

^b ^superscripts indicate p < 0.001 (Kruskal-Wallis) between all three areas.

Similarly, for ewes, the proportion of flocks with more than three treatments per year in the coastal, inland and northern area was 10.6%, 2.2% and 0.0%, respectively. The use of benzimidazoles in sheep flocks decreased significantly (p < 0.001) from 73.5% of the flocks in 2005 to 64.9% in 2007. During the same period, there was a significant increase (p < 0.001) in the use of macrocyclic lactones in the sheep flocks from 14.3% of the flocks to 23.4% (Table 4). Five sheep flocks used no anthelmintic treatment at all during the period 2005-2007.

**Table 4:** Type of anthelmintics used during 2005 - 2007 in sheep and goat flocks in Norway (percentage).

Type of anthelmintics	Sheep			Goat		
	2005	2006	2007	2005	2006	2007
	
	*n *= 551	*n *= 561	*n *= 587	*n *= 205	*n *= 203	*n *= 189
Benzimidazoles (BZ)	73.5^a^	66.3^a^	64.9^a^	51.3^a^	46.6^a^	42.3
Macrocyclic lactones (ML)	14.3^b^	21.1^b^	23.4^b^	32.9^b^	36.0^b^	39.1
Tetrahydropyrimidines (THP)**	4.4	2.1	0.9	-	-	-
BZ + ML	6.7	9.4	9.7	7.9	8.1	8.3
No treatment	1.1	1.1	1.1	7.9	9.3	10.3

Total	100	100	100	100	100	100

^a, b ^different superscripts in the same column indicate a significant difference (p < 0.001, Kruskal-Wallis) between BZ and ML within the years.

**In the anthelmintic class Tetrahydropyrimidines only the label "Exhelm^®^, Pfizer, (Morantel)" was used. This class has not been registered in Norway after 2006.

#### 1.5 Drug alternation

The proportion of farms using both benzimidazoles and macrocyclic lactones the same year was significantly higher (p < 0.001) in the coastal area compared to the inland and northern area (Table 5). The two anthelmintics were used alternately and not at the same time. More flocks choose macrocyclic lactones in the inland and northern area than in the coastal area. The use increased from 15.5% and 16.0% of the flocks in the inland and northern area, respectively, in 2005 to 29.4% and 28.0% of the flocks in 2007. In the period 2005 - 2007, 46.3% of the sheep flocks never changed the anthelmintic class, whereas 16.8% of the flocks changed the anthelmintic class two times or more.

**Table 5:** Type of anthelmintics used in the different areas during 2005 - 2007 in sheep flocks (percentage) in Norway (see also Table 4).

Area	Year	BZ	ML	THP	BZ/ML	No treatment
Coastal	2005	71.9	13.6	2.2	11.4^a^	0.9
	2006	65.9	15.1	2.2	16.4^a^	0.4
	2007	65.2	18.5	0.0	15.9^a^	0.4
Inland	2005	73.8	15.5	7.1	2.4 ^b^	1.2
	2006	62.9	24.7	5.6	5.6 ^b^	1.1
	2007	62.4	29.4	1.2	9.3 ^b^	1.2
Northern	2005	74.5	16.0	6.4	3.2 ^b^	0.0
	2006	66.0	30.9	1.1	2.1 ^b^	0.0
	2007	71.0	28.0	0.0	1.1 ^b^	0.0

^a,b ^p < 0.001(Chi-Square) between coastal area compared with inland and northern area regarding use of both BZ and MLs for a given year.

### 2. Goat flocks

#### 2.1 General information

In the dairy herds, the Norwegian dairy goat was the only breed present. Kashmir and Angora were the two most common fiber goat breeds. The time of turnout was mainly in Mid-May (90.3% of the flocks), with an average grazing period of five to six months (Table 1). The main kidding period was from December to March, and 89.4% of the kids were two months or older at turn out. Co-grazing with cattle or sheep was practiced by 74.6% of the flocks (data not shown).

#### 2.2 Drenching time and frequency

On 84.7% of the dairy goat farms, the anthelmintic was administered during the dry period (Table 2). The yearly mean drenching rate in both kids and adult goats was one time per year. However, in 2007 10.3% of the goat flocks did not receive any anthelmintic treatment at all.

#### 2.3 Dose-estimation

To determine the anthelmintic dose, 85.1% of the goat farms used visual appraisal of weight for a common weight estimation for the entire flock. In 8.3% of the goat flocks, the anthelmintics were administered by a veterinarian using a subcutaneous injection of macrocyclic lactones. However, there was no information regarding what kind of weight estimation that had been used in these flocks. In 38.1% of the goat flocks, the farmers never checked the accuracy of their drench gun.

#### 2.4 Drug alternation

Benzimidazoles were the most common anthelmintic class used in goats (Table 4). The proportion of flocks using macrocyclic lactones increased from 32.9% in 2005, to 39.1% in 2007. In the same time period, the number of goat flocks using no anthelmintic treatment increased from 7.9% to 10.3% (Table 4). There were no differences between the three areas regarding the use of different anthelmintic classes or combinations in the goat flocks.

## Discussion

In the present study, the response rate was above 50%. The response rate for sheep flocks were 42.7% in flocks with 20-100 ewes, and 58.7% in flocks with more than 100 ewes. Compared to the total number of sheep flocks in Norway, flocks with less than 100 winter fed ewes were underrepresented. Similar surveys have been based on a lower response rate, ranging from 15% to 24% [16,25,28].

### Drenching time

The maintenance of a pool of susceptible parasites not exposed to the drug as free-living stages in the environment or as adults in un-drenched animals, i.e., worms in *refugia*, seems to play an important role in the prevention of anthelmintic resistance in ruminants [13]. The apparent usefulness of maintaining worms in *refugia *influence the different ways of controlling the gastrointestinal nematode population in grazing livestock. In the sheep and goat flocks in this survey, the decision to use anthelmintics was not evidence-based using clinical indicators such as scouring or weight loss or coproscopical examinations, and thus no targeted selective treatment was performed. Actually, in 57.4% of the sheep flocks, the main triggering factors for treating was weather and climatic observations during the grazing season, pasture management and the experience from earlier years, and the whole group of animals (young or adults) was treated. In general, the anthelmintic treatment seemed to have been based mainly on established routines that in previous years had given an apparently economical and sustainable livestock production.

In the present study, 35.8% of the sheep farmers combined the anthelmintic treatment with a change of pasture, i.e., a dose-and-move strategy. Such practices were especially used for ewes and lambs before transporting them to the common grazing areas in the forest or mountains during the summer months. The dose-and-move strategy in small ruminant flocks has also been noticed as a common practice in other European countries [25,31-33]. However, moving drenched sheep onto pastures with a low level of parasite contamination increases the risk for AR [34,35]. The role of the mountain and forest pastures as potential *refugia *for the gastrointestinal nematodes is thus a key question. Indeed, the cold and long winter period, which probably allows a limited survival of overwintering infective larvae, in combination with anthelmintic treatment prior to moving the animals, might represent a significant selective pressure on the worm population [36,37]. Only *Teladorsagia circumcincta *and *Nematodirus battus *among the most pathogenic nematodes are regarded as being capable of overwintering on pasture in Norway [38]. However, the low animal density decreases the probability of finding a new host for the nematodes on mountain pasture. The duration from September until June next year, when the animals are re-entering these pastures contribute to decrease the worm burden [39]. On the other hand, some nematode populations, such as *Haemonchus contortus*, are unable to survive as overwintering larvae under Nordic conditions and refugia as free-living stages from year to year may be considered as virtual [40]. This epidemiological trait of *H. contortus *has led to consider a possible eradication of this worm from farms by treating all the animals when housing with an anthelmintic drug achieving a high efficacy against inhibited larvae [40]. In our work, anthelmintic treatment was given to ewes either at housing or during the housing period in 66% of the sheep flocks. As far as *H. contortus *is concerned, this practice represents a real threat for AR to emerge.

### Under-dosing

A poor drenching practice can result in under-dosing of a drug and select for AR [41,42]. Not only incorrect estimation of animal live-weight, but also incorrect calibration of drench guns can cause under-dosing. To ensure a correct dose, one has to estimate the weight as accurately as possible [2], preferably by an individual weighing of each animal. Weighing the heaviest animal before drenching all animals with a slightly over-calculated dose can also be considered as an appropriate way to ensure correct anthelmintic dose.

In 78.6% of the sheep flocks, visual appraisal of weight based on knowledge and experience was used for calculating the anthelmintic dose. In the goat flocks, 85.1% used visual appraisal for weight estimation. Similar results were found in Slovakia and France, where visual weight estimation was used in 87.8% and 100.0% of the small ruminant flocks, respectively [31,32]. If weighing each animal or only the heaviest animals, were considered as the only acceptable method for calculating the dose, only 8.3% of the sheep flocks and 2.5% of the goat flocks in our survey had an appropriate dose calculation.

The present study showed that 90% of the anthelmintics used in sheep and goat farming in Norway were administrated orally. It is known that the efficacy of oral drenches, in particular benzimidazoles, depends partly on the extent of oesophageal groove closure, and that this partial or complete rumen bypass is a very frequent phenomenon at least in goats [43]. The issue regarding the use of specific anthelmintic dose rates for goats was not investigated in this survey. However, earlier studies have shown that goats metabolize anthelmintics faster than sheep, in particular as regards the benzimidazoles, and this has led to an advice of using higher anthelmintic dose rates for goat compared to sheep [44]. In Norway, only fenbendazole (Panacur vet., Intervet) has been licenced for goats, and then at the same dose rate as for sheep (5 mg/kg). This dose is clearly inappropriate for goats [45]. As a result, in some countries such as France, specific recommendations have been added in 2008 in the "Summary of Product Characteristics" of several benzimidazole compounds (Chartier, personal communication). In Norway, the use of the sheep dose for goats seems most common (Leine, personal communication). Eprinomectin (Eprinex vet, Merial) has been used by goat farmers the last years although this product has been licenced only for cattle in Norway.

In addition, the drench gun was never controlled in 27.1% of the sheep flocks and in 38.1% of the goat flocks. An inaccurate drench gun may also contribute to under-dosing of the anthelmintics [46].

### Frequency

A high treatment frequency is considered as a major risk factor for the development of anthelmintic resistance [47-49]. The mean drenching rates in lambs and ewes in Norway was 2.5 and 1.9 times per year, respectively. These figures are of a similar magnitude to those reported elsewhere in northern Europe. Thus, in Denmark, Maingi et al. reported a mean drenching rate in lambs and ewes of 1.9 and 2.3 per year, respectively [25]. In Scotland and England, the mean drenching rate ranged from 2.2 to 4.4 annual treatments per lamb, respectively [28,50]. However, in the present work, the drenching frequency in lambs and ewes was significantly higher in the coastal area (3.4 and 2.2, respectively) than in the inland and northern area.

It is unknown if the high drenching frequency in the coastal area could be a sign of a higher parasite challenge or of anthelmintic resistance. High treatment frequency may be due to the longer grazing season and a more favourable environment for larval development and survival in the coastal area. Recent reports from the UK indicates that *H. contortus *and *T. circumcincta *are able to establish further north than earlier reported [51]. In Norway, the coastal areas have a similar climate as found in Scotland and England and represent a more favourable location for nematode infections compared to northern areas. There is also a prolonged grazing period near the farm on cultivated pastures in the coastal area compared to the inland and northern area [52]. Normally, the animal stocking rate on the cultivated pastures in the coastal area is high in the spring compared with the inland and northern area [53]. However, since the treatment frequency is not based on clinical signs or laboratory examinations, the necessity of frequent drenching has to be elucidated.

### Drug alternation

In 83.2% of the total number of sheep and goat flocks, no changes of anthelmintic class were done in the investigated period. Benzimidazoles (BZ) were the major anthelmintic class used in sheep and goat flocks (Table 4). However, there was a slight increase in the use of macrocyclic lactones (ML) in all areas. In general, the change to MLs use was more dominant in sheep flocks in the inland and northern areas. The recent emphasis on drug alternation from Norwegian animal health organizations can have resulted in an increased use of MLs. Other reasons for the increased use of MLs can be their persistent activity and their better efficacy on inhibited larval stages of gastrointestinal nematodes. MLs also have an effect on ectoparasites and the conveniences of using pour-on products, e.g. eprinomectin. This probably explains the common use of MLs in goat flocks compared to sheep flocks. A reduced drug efficacy could also lead to a change of anthelmintic drug class. So far, only sporadic cases of AR in small ruminant flocks have been reported in Norway [12]. Thus none of the farmers in the survey have reported reduced anthelmintic effect.

### Norway compared with other European countries

The worm control management and use of anthelmintics in Norway do not fundamentally differ from general practices in Denmark, France, Slovakia and the UK [25,28,29,31,32]. To some extent, inaccurate dose calculations and a dose-and-move strategy seem to occur in all these countries. However, as a result of having only two anthelmintic classes on the marked in Norway, drench alternation is not a common practice. Regarding the drenching frequency, our results in lambs are similar to what have been reported from UK. [28]. In contrast, the common use of mountain and forest pasture during the summer months combined with a low stocking rate is probably the main peculiarity of Norwegian sheep farming.

## Conclusions

The present study has shown that the anthelmintic drenching routines used in small ruminants in Norway may contribute to the development of AR. Over 90% of the sheep and goat flocks had insufficient weight estimation for calculating correct anthelmintic dose. This combined with few farmers controlling their drench gun represents a high risk for under-dosing anthelmintics in these flocks. A high risk was also suggested by a high treatment frequency, especially in lambs in the coastal area, a lack of anthelmintic class rotation and a common use of a dose-and-move practice. The early detection of AR may be quite difficult [50,54]. In order to avoid or slow down the emergence of AR, correct use of anthelmintics, on-farm information of gastrointestinal parasite burdens and knowledge regarding how to maintain parasites in *refugia *have to be implemented in the worm control management. This includes focus on dose rate, anthelmintic class alternation, treatment frequency, stocking rate and new treatment strategies, such as targeted (selective) treatment in combination with faecal egg counts. Giving the farmers the right information regarding worm control is a key stone in preventing anthelmintic resistance. Since the distribution of anthelmintic resistant nematodes in Norway is unknown a national surveillance program for AR detection should be established.

## Competing interests

The authors declare that they have no competing interests.

## Authors' contributions

AVMD, CC, BG, NL, SV, OØ and SS initiated and designed the study. AVMD performed the questionnaire and recorded the data. AVMD and OØ performed the statistical analysis. AVMD and SS drafted the manuscript. All authors read and approved the manuscript.
